# Surgical Management of Adult Brainstem Gliomas: A Systematic Review and Meta-Analysis

**DOI:** 10.3390/curroncol30110709

**Published:** 2023-11-07

**Authors:** Tamara Ius, Giuseppe Lombardi, Cinzia Baiano, Jacopo Berardinelli, Andrea Romano, Nicola Montemurro, Luigi Maria Cavallo, Francesco Pasqualetti, Alberto Feletti

**Affiliations:** 1 Neurosurgery Unit, Head-Neck and NeuroScience Department, University Hospital of Udine, 33100 Udine, Italy; tamara.ius@gmail.com; 2 Department of Oncology 1, Veneto Institute of Oncology IOV-IRCCS, 35128 Padua, Italy; lombardi.giuseppe.lg@gmail.com; 3 Division of Neurosurgery, Department of Neurosciences, Reproductive and Odontostomatological Sciences, Università degli Studi di Napoli Federico II, 80131 Naples, Italy; baianocinzia@gmail.com (C.B.); luigimaria.cavallo@unina.it (L.M.C.); 4 Department of Neuroradiology, NESMOS S.Andrea Hospital, University Sapienza, 00189 Rome, Italy; andrea.romano@uniroma1.it; 5 Department of Neurosurgery, Azienda Ospedaliero Universitaria Pisana, 56123 Pisa, Italy; nicola.montemurro@unipi.it; 6 Department of Oncology, University of Oxford, Oxford OX3 7DQ, UK; francep24@hotmail.com; 7 Department of Neurosciences, Biomedicine, and Movement Sciences, Institute of Neurosurgery, University of Verona, 37129 Verona, Italy; alberto.feletti@univr.it

**Keywords:** brainstem glioma, surgery, radiotherapy, radiosurgery, biopsy, intraoperative neurophysiological monitoring, meta-analysis, extent of resection, survival

## Abstract

The present review aims to investigate the survival and functional outcomes in adult high-grade brainstem gliomas (BGSs) by comparing data from resective surgery and biopsy. MEDLINE, EMBASE and Cochrane Library were screened to conduct a systematic review of the literature, according to the PRISMA statement. Analysis was limited to articles including patients older than 18 years of age and those published from 1990 to September 2022. Case reports, review articles, meta-analyses, abstracts, reports of aggregated data, and reports on multimodal therapy where surgery was not the primary treatment were excluded. The ROBINS-I tool was applied to evaluate the risk of bias. Six studies were ultimately considered for the meta-analysis. The resective group was composed of 213 subjects and the bioptic group comprised 125. The analysis demonstrated a survival benefit in those patients in which an extensive resection was possible (STR HR 0.59 (95% CI 0.42, 0.82)) (GTR HR 0.63 (95% CI 0.43, 0.92)). Although surgical resection is associated with increased survival, the significantly higher complication rate makes it difficult to recommend surgery instead of biopsy for BSGs. Future investigations combining volumetric data and molecular profiles could add important data to better define the proper indication between resection and biopsy.

## 1. Introduction

Brainstem glioma (BSG) represents a rare genetically and radiographically heterogeneous group of tumors most frequently found in the pediatric population, where, unlike other primary brain tumors, they show a worse prognosis than their adult counterparts [1,2,3,4,5,6,7,8,9]. Adult BSGs are less frequent than in children, representing only 1–2% of all brain malignant tumors, making them less well characterized due to their rarity [1,2,3,4].

Disease evolution in the adult population seems to have a less aggressive clinical course and behavior than in the pediatric one, with survival rates ranging from 1 to 7 years [1,2,4,6,8,9].

Prognosis and treatment depend upon clinical, histologic, imaging, and molecular features [4,8,9,10]. Recent advancements in the fields of genetics and histopathology have shown that the genomic profile of adult BSGs significantly differs from that of both its pediatric counterpart and adult supratentorial gliomas. The identification of the H3K27m mutation, introduced in the WHO classification of 2016, has been proposed as a significant diagnostic and prognostic factor, opening the target therapy scenario [4,10].

Despite the genetic and molecular discoveries, evidence regarding the treatment options is limited and conflicting in the current literature [4,5,7,11,12,13,14,15,16,17,18,19]. The low incidence and wide heterogeneity amongst different clinical series, in terms of classification and treatment options, make it difficult to define an adequate standardized therapeutic approach in these patients.

For a long time, surgery was considered an absolute contraindication for BSG, mainly due to the structural, functional, and vascular complexity of the brainstem [11,12,13,14,15,16,17,18,19].

**Figure 1:**
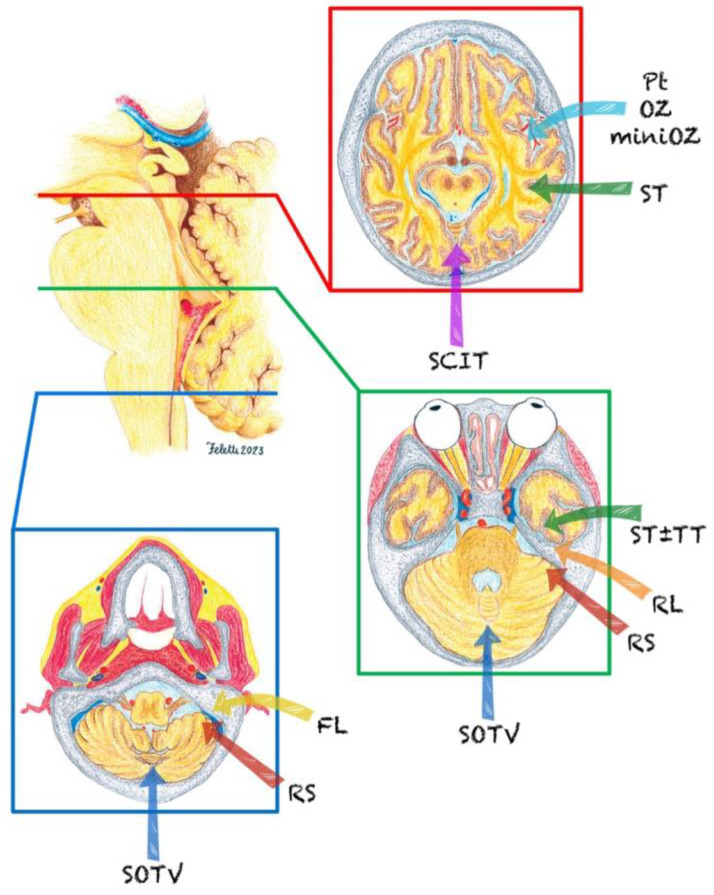
Surgical approaches to the brainstem. Main surgical routes used to approach midbrain (red), pons (green), and medulla (blue). FL = far lateral; OZ = orbitozygomathic; Pt = pterional; RL = retrolabyrinthine; RS = retrosigmoid; SCIT = supracerebellar infratentorial; SOTV = suboccipital trans telovelar; ST = subtemporal; TT = trans temporal.

For focal exophytic lesions, surgery is increasingly accepted as a primary mode of treatment, with multiple surgical approaches proposed, according to tumor localization (Figure 1). Conversely, in the case of the highly malignant entity known as diffuse midline glioma (DMG), the nonsurgical strategy is well established, while the role of surgery for lesions between these two extremes remains unclear and controversial [5,19]. The biopsy-derived histopathological materials present diagnostic challenges due to intratumoral heterogeneity, with the risk of sampling bias and the small amount of tissue that restricts additional molecular workup.

The aim of this systematic review and meta-analysis was to investigate the overall survival (OS) and functional outcomes in adult BSG patients who underwent surgical resection in comparison with those treated with biopsy and adjuvant treatments.

## 2. Materials and Methods

### 2.1. Study Design

The present study is a systematic review of the literature, consistently conducted according to the preferred reporting items for systematic reviews and meta-analyses (PRISMA) statement guidelines [20]. The investigation followed a prespecified protocol which was registered on PROSPERO (PROSPERO 2023 CRD42023402271).

### 2.2. Review Question

The review questions, according to the PRISMA statement, were formulated following the PICO (P: patients; I: intervention; C: comparison; O: outcomes) scheme, as follows:

In newly diagnosed adult high-grade BSGs (P), has the extent of resection (I) been revealed as effective when compared to biopsy (C) in terms of risk of mortality assessed as hazard ratio?

The secondary aims of this review were to investigate possible relationships between adjuvant therapy (chemotherapy and radiotherapy) or lesion location and mortality.

### 2.3. Inclusion and Exclusion Criteria

Analysis was limited to articles that included patients older than 18 years of age at the time of diagnosis and those published in 1990 or later, given the rise of magnetic resonance imaging (MRI). Comparative papers in which patients underwent either biopsy or surgical resection of histologically confirmed brainstem gliomas were included in the following review.

Papers regarding surgical management of pediatric brainstem gliomas were not included in the following study. Case reports, review articles, meta-analyses, abstracts, reports of aggregated data (different location or pathology), and reports on multimodal therapy where surgery was not the primary treatment were excluded. In addition, exclusion criteria encompassed languages other than English, noncomparative studies, and nonreported quantitative data for analysis. Duplicated papers were excluded from the screening.

### 2.4. Search Strategy

Four different medical databases (MEDLINE, EMBASE, Mendeley, and Cochrane Library) were screened in order to conduct a systematic review of the literature, according to the PRISMA statement [20], evaluating the role of surgery in adult BSG patients.

Records were searched for pertinent studies from 1990 to September 2022. We reviewed all abstracts of English-language articles containing the following keywords alone or in combination (using the Boolean operator “and”): “glioma”, “gliomas”, “brainstem”, “adult”, “surgery”, “biopsy”, “microsurgery”, “resection”, and “treatment”. Each article of interest was marked for further review. The references listed in each paper were also reviewed for pertinent articles. The review of the titles and abstracts was conducted by two investigators (C.B., J.B.). For studies that warranted full-text review, the same two reviewers evaluated each study independently. Any discordance in the screening process was solved by consensus of four senior authors (T.I., G.L., A.F., F.P). From each study, single case records including demographic data, time to treatment, symptoms at diagnosis, lesion characteristics, and treatment settings as well as clinical, radiological, and oncological outcomes of interest were extracted. All extracted data were audited by the two independent authors for accuracy and completeness.

### 2.5. Outcome Measurements

The following data were extracted from the included papers: general identification information (author, title, journal, and date of publication); intervention characteristics; main clinical, radiological, and anatomopathological characteristics; HRs with corresponding 95% Cis; overall survival, defined as the time from surgery to patient death; and all reported postoperative complications, defined as intracerebral bleeding, motor deficit, speech disorders, decreased upgazed, subgaleal hematoma, cranial nerve palsy, and cerebrospinal fluid (CSF) leak.

### 2.6. Quality Scoring

The ROBINS-I tool was applied to evaluate the risk of bias (RoB) in nonrandomized controlled trials (non-RCTs), detected by the screening process [21,22].

The overall RoB was categorized as critical, serious, moderate, low, or with no available information. The RoB assessment was performed independently by two investigators (C.B. and J.B.), and any discrepancies were resolved by a third author (T.I.).

### 2.7. Statistical Analysis

Categorical variables were reported as absolute numbers and percentages, whereas continuous variables were reported as median value ± standard deviation. Chi-square of Fisher’s exact tests were used when appropriate to compare categorical variables. For time-to-event variables, the effect of the treatment for each individual study was expressed as a hazard ratio (HR) and 95% CI. For the meta-analysis, the HRs and their standard errors (SEs) were transformed into their log counterparts, applying the inverse variance method, and then back into the HR scale [23]. The raw data were entered into Microsoft Excel (Version 16.63.1 for Mac). Statistical analyses were performed via R (version 4.0.2; The R Foundation for Statistical Computing, Vienna, Austria) and RStudio (version 1.2.1335), with a 2-tailed *p* < 0.05 considered significant. Hazard ratios were compared using the “metafor” function in R. Whether random effects or fixed effects should be used was decided by the I^2^ tests. The “Forest” and “Funnel” functions in R were used for the respective plot with subgroup analysis.

## 3. Results

### 3.1. Included Studies and Patients

**Figure 2:**
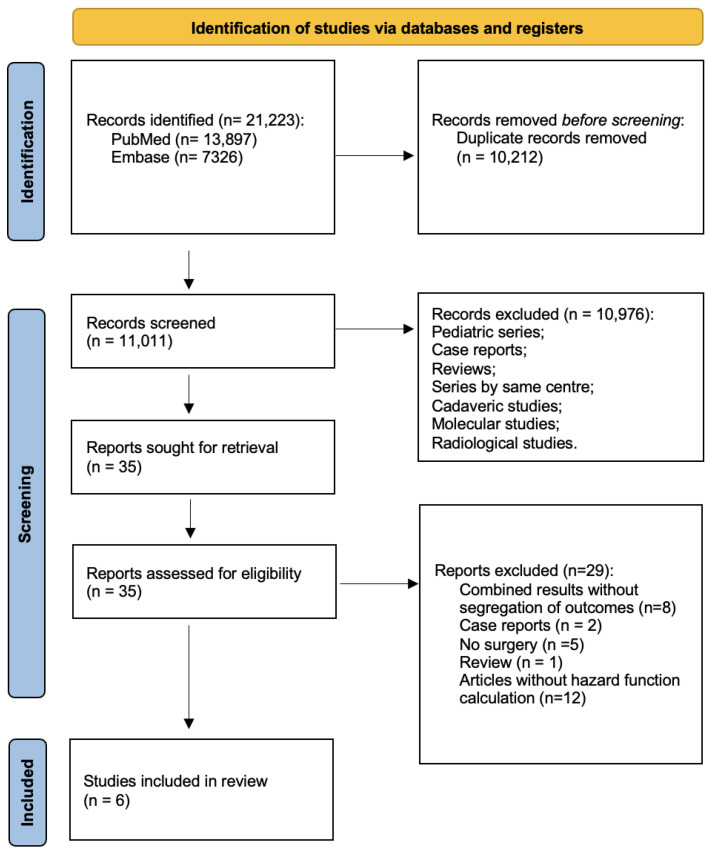
PRISMA flow chart of search strategy divided by identification, screening, eligibility, and inclusion.

The search strategy is summarized in Figure 2. After excluding with reason 29 manuscripts (Supplementary Table S1), 6 retrospective papers were included for systematic review [7,13,14,15,16,17] (Figure 2) and meta-analysis. The low number of included studies with retrievable data regarding low-grade gliomas and the insufficient sample size did not allow a direct comparison between resection and biopsy outcomes among this subgroup.

Overall, 213 patients with high-grade BSGs were included in the surgical resection group (RG, experimental group), and 125 in the biopsy group (BG, control group).

### 3.2. Quality of Studies

All of the included studies [7,13,14,15,16,17] were retrospective, single center, and rated “moderate” to “low” risk of bias (Supplementary Figure S1).

### 3.3. Clinical Results

The baseline demographical, anatomopathological, and surgical characteristics of the study population are summarized in Table 1. Histopathological grading was reported in all studies, while the extent of resection was specified only in four out of the six studies. Main clinical and radiological characteristics as well as additional anatomopathological and surgical data are presented in Supplementary Tables S2 and S3. Three of the included papers reported the localization of the lesions, with the pons most affected by the tumor (63.8%). Multifocal presentation, categorized as concomitant involvement of multiple brainstem departments, was present in 36.2% of the reported cases. It was not possible to assess the surgical approach of choice used in the RG.

### 3.4. Surgical Resection

**Figure 3:**
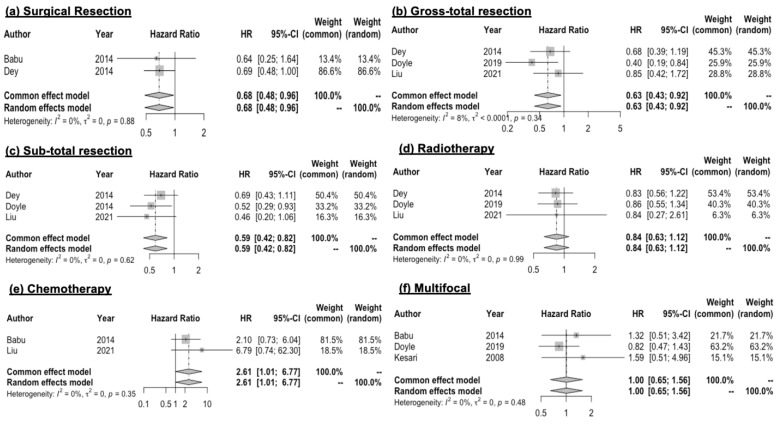
Forrest plot–hazard ratio (HR) [7,13,14,15,17]. The HR of all included patients demonstrated that there was a significant advantage in patients undergoing (**a**) surgical resection in brainstem high-grade gliomas (HGG). Advantage in overall survival was also observed in subgroups of patients undergoing (**c**) subtotal resection or (**b**) gross-total resection. The HR of all included patients demonstrated that there was not a statistically significant advantage in patients undergoing (**d**) adjuvant radiotherapy for brainstem high-grade glioma. An increased risk of mortality was found among patients undergoing (**e**) adjuvant chemotherapy for brainstem high-grade gliomas (HGG), while (**f**) multifocal presentation of brainstem high-grade gliomas was found to have an HR of 1, showing no effect on patient overall risk.

Quantitative data concerning surgical resection and OS were reported in four of the included papers [7,13,14,15]. The analysis of the metadata revealed low heterogeneity among the included studies and a statistically significant advantage in terms of mortality for patients with brainstem high-grade gliomas treated with surgical resection when compared to the biopsy control group. Survival advantage was evident in the whole surgical sample (HR: 0.68; 95% CI 0.48–0.96). These data were also confirmed in both subgroups undergoing subtotal resection (STR HR 0.59 (95% CI 0.42–0.82)) and gross-total resection (GTR HR 0.63 (95% CI 0.43–0.92)) (Figure 3a–c).

### 3.5. Adjuvant Radiotherapy

The quantitative data on adjuvant radiotherapy for high-grade BSGs were reported in three [7,15] of the included studies, with a total of 271 patients in the experimental group and 121 in the control group. The HR of all included patients did not demonstrate a statistically significant advantage for patients undergoing adjuvant radiotherapy for high-grade brainstem gliomas (HR: 0.84; 95% CI 0.63–1.12) (Figure 3d).

### 3.6. Adjuvant Chemotherapy

The quantitative data on adjuvant chemotherapy were reported in two [7,15] of the included studies, which were based on a total of 34 patients in the experimental group and 52 in the control group. Only one study specified the initial chemotherapy adjuvant treatment, with most of the population receiving concurrent temozolomide and half of the study group receiving other chemotherapeutics on tumor progression. The HR of all included patients demonstrates that there was an increased risk of mortality in patients undergoing adjuvant chemotherapy for brainstem high-grade gliomas, even though a high heterogeneity must be considered (HR: 2.61; 95% CI 1.01–6.77) (Figure 3e).

### 3.7. Multifocal Localization and Surgical Resection

Surgical resection associated with localization was reported in three [7,14,17] of the included studies. There was not any statistically significant difference when comparing hazard ratios between patients with or without multifocal presentation of brainstem high-grade gliomas (HR: 1.00; 95% CI 0.65–1.56) (Figure 3f).

### 3.8. Level of Precision among Studies

The funnel plot shows that all included studies have a moderate level of precision to investigate the surgical effectiveness of the resection (Supplementary Figure S2).

Publication bias was assessed using the Sterne and Egger method and is displayed in a funnel plot. No clear evidence of publication bias resulted from this analysis.

### 3.9. Postoperative Complications Analysis

The postsurgical complications rate for BG was 10.5%. For RG, the frequency was 35.5%. The difference between the rate of complication in RG and BG was found to be statistically significant with *p* = 0.009 (Table 2).

## 4. Discussion

Adult BSGs are heterogeneous lesions in terms of molecular features, tumor growing pattern, and survival [4,6,8,9,11]. The rarity of the disease, in association with the heterogeneity in tumor histology and treatment options described amongst the case series, limits the group analysis and thus the definition of the best decisional treatment algorithm.

For a long time, BSGs were not considered for surgery, primarily due to the intricate vascular and anatomic–functional anatomy of this region. Continuous technical improvements in the operative technique have allowed surgery to become a feasible therapeutic option in selected cases [4,5,7,11,12,13,14,15,16,17,18,19]. In addition, the debate over the importance of biopsy has been renewed by the widespread use of intraoperative monitoring (IOM) to functionally detect safe entry zones and by the increasing demand for molecular profile definition prior to initiating any treatment option [24,25].

Considering the literature discrepancies, this investigation aims to analyze the safety of surgical procedures and to explore the role of surgery in terms of mortality risk, making the series data as uniform as possible by using the meta-analytic approach.

### 4.1. Extent of Resection and Survival

The management of BSG remains controversial, with increasing evidence supporting surgical resection as the primary treatment for selected cases [5,7,17]. Recent evidence suggests that surgical resection may be a viable primary treatment option for selected cases of brainstem glioma, especially those with significant clinical symptoms, mass effect, tumor progression, or hydrocephalus [5,19]. The role of radical surgery is well established and widely documented as the first step in supratentorial HGG management [26,27,28,29]; however, it has remained poorly documented in the setting of BSG [2,3,6,10,12,14,16].

Babu et al. analyzed a retrospective surgical series of 34 adult BSGs, showing that the RG had a median survival nearly two times longer than the BG. A significant difference between these two groups was not, however, detected, likely due to the highly documented variability in the molecular profiles between groups [7]. The authors also analyzed survival in relation to tumor localization, reporting how tumors in the midbrain show poorer survival when compared with lesions located in the pons (5.9 vs. 42.1 months, *p* = 0.006) and found a slight, although not significant, survival benefit with surgical resection for midbrain and pontine BSGs [7]. It is necessary to contextualize this finding in light of the small sample size and to further assess it in subsequent research in order to precisely identify BSG subcategories that would benefit more from a surgical approach.

In a later investigation based on 240 adult patients, Dey et al. found that younger age and lower-grade histology were the strongest prognosticators, while surgical intervention trended towards a significant association with benefit in survival [13].

This finding was corroborated in an independent multicenter analysis of 47 BSGs by Rigamonti and colleagues, where a univariate analysis revealed that tumor grade was the only factor with a statistically significant impact on overall survival. Furthermore, younger age, better performance status, and total/subtotal resection showed a trend towards prolonged survival for both LGG and HGG. The survival benefit was also observed in different resective classes (GTR versus STR or STR versus biopsy) when compared to each other [12].

Doyle et al. [14] analyzed 103 adult BSG patients, showing a survival benefit when surgery was more aggressive. This result was further accentuated within the groups where different EORs were achieved, with partial resection having an HR of 0.32 (*p* = 0.006) and total/GTR having an HR of 0.24 (*p* < 0.001).

Recently, Faulkner and colleagues [19] conducted a study on a larger patient population with HGG BSG and concluded that both GTR and STR surgeries are feasible options for exophytic lesions. Their research confirms that a comprehensive extent of resection (EOR) can improve survival even with different levels of resection.

Only one of the studies included in this meta-analysis [17] tried to correlate the presence of an exophytic component and survival outcomes, finding a nonstatistically significant worse prognostic trend (HR 1.80, 95% C.I. 0.53–6.08). Even though the transgression of normal brainstem parenchyma is intuitively associated with a higher degree of morbidity and worse outcomes, the implementation of the validated safe entry zones [30,31] in the hands of expert brainstem surgeons in high-flow centers could explain this finding and potentially increase the number of patients eligible for surgery. Overall, these studies show that surgical resection is beneficial for focal BSG; however, it is difficult to compare the results of different investigations because there is no stratification based on tumor grade or molecular markers.

Biopsy is assuming a central role in nonresective cases as the histo-molecular definition is an emerging requirement for administering targeted personalized treatments based on specific mutations. However, it should be noted that for the majority of the investigation, decisions about treatment were made using MRI data because brainstem stereotactic biopsy is frequently regarded as being too risky due to its potential for morbidity (transient or permanent) or mortality rates as high as 28%, 9%, and 4%, respectively [32,33,34].

But as Kickingereder et al. recently showed in a well-designed meta-analysis, stereotactic biopsy is a valuable, reliable, and secure diagnostic technique, even for midline diffuse glioma [18], with a diagnostic success rate of 96.2% and rates of permanent morbidity and mortality of 1.7% and 0.9%, respectively.

Additionally, the endoscopic ventricular approach has also been proposed as an alternative to obtain bioptic samples from brainstem lower-grade gliomas with obstructive hydrocephalus, with encouraging outcomes in terms of safety and diagnostic accuracy [35,36].

### 4.2. Radiotherapy

Radiation therapy remains a cornerstone of treatment protocols for adult BSGs. It is important to consider that the six selected studies did not primarily focus on evaluating the role of radiotherapy, and comprehensive information regarding the doses and fractionations utilized was not provided in all the papers.

Furthermore, the wide time span over which patients were treated, the differences in prescribed doses, and the techniques used make it difficult to assess the impact of radiotherapy. In the study by Babu et al., almost all 34 patients received radiotherapy treatment from 1988 to 2011, and doses ranged from 5580 cGy to 6300 cGy. In the study by Kesari et al., the radiotherapy dose ranged from 2400 cGy to 9180 cGy (150–200 cGy/session). In that study, 88% of the patients showed an initial response to radiotherapy, and all relapses were within the treatment range.

Despite the wide variation in postoperative radiotherapy response rates reported in the literature, our meta-analysis and all the included studies failed to provide a statistical survival benefit. One possible explanation could be related to selection bias in patients undergoing radiation therapy, whereby a considerable proportion of patients are selected for this treatment after undergoing a biopsy or partial resection [37]. This might also suggest a heightened resistance and a more aggressive biological behavior of BSG in comparison to their supratentorial counterparts.

In conclusion, our results are consistent with those obtained by a large-scale population-based study from the National Cancer Database where RT alone compared with no treatment was not statistically associated with a reduction in mortality risk (HR 1.25, 95% C.I. 0.82–1.89) [38]. A survival benefit was found only when RT was associated with chemotherapy (HR 0.67, 95% C.I. 0.46–0.98), as also confirmed by Liu et al. [15].

Additionally, it is crucial to acknowledge the potential substantial impact on survival due to significant morbidity resulting from radiation treatment in this highly eloquent region.

### 4.3. Chemotherapy

Systemic treatments for adult BSG have been primarily explored through a limited number of single-arm retrospective studies [39]. Chemotherapeutic agents are often combined with radiation therapy to capitalize on their synergistic impact. However, the absence of established guidelines has led to a wide array of treatment protocols, particularly after surgical resection or during disease progression.

Kesari et al. reported the application of experimental chemotherapy protocols (etoposide, paclitaxel, tamoxifen, hydroxyurea, and radiolabeled anti-EGF antibodies) after initial treatment failure in patients with adult BSG. Results showed no survival benefits and a decrease in both median OS (78 vs. 108 months for treated vs. untreated) and five-year survival rate (54.0 vs. 61.0%) [17].

DeWire et al. conducted a phase I/II trial to identify the efficacy of ribociclib (CDK4/6-inhibitor) in newly diagnosed DIPG patients as an adjuvant monotherapy after radiotherapy. Results showed a moderate increase in median OS (16.1 months) but presented some safety concerns, with an increased tumor necrosis volume in almost half of the study population [40].

El-Khouly et al. conducted a phase I/II trial, testing a combination of bevacizumab, irinotecan, and erlotinib in patients with progressive DIPG. The trial showed an extended median overall survival of 13.8 months compared to historical control cohorts [41].

Additional research is needed to elucidate the efficacy of radiation therapy and chemotherapy for adult BSG. However, it is important to highlight that the existing literature supports the application of a combined radiotherapic/chemotherapic protocol.

### 4.4. Complications

The most common complications in BSG surgery included brainstem edema, respiratory dysfunction, and cranial nerve dysfunction [4,6,9].

The RG had a higher complication rate than the BG, as expected [7,16,17,18]. Regarding surgical safety, first, Teo and Siu in 2008 demonstrated that the degree of resection was not associated with long-term poor outcomes at 6 months [5].

Kesari et al. in a retrospective, well-designed analysis of 101 adult BSGs found complication rates of 29% and 40% in the BG and RG, respectively [17]. In this meta-analysis, the complication rate was 10.5% BG and 35% in RG. This result may be explained by the less invasive nature of the biopsy, which, however, is not without risk, considering the depth and anatomical complexity of this surgical area.

Unfortunately, only some of the studies included in this meta-analysis described the postoperative deficits [7,16,17]. In addition, surgical series rarely stratify the postoperative deficits within the different classes of resection. The lack of accuracy in describing the postoperative deficits may result in an overestimation of deficits related to the RG.

It should also be considered how tumor growth in the brainstem itself presents with a high degree of morbidity. In the included studies, the authors observed cranial nerve dysfunction rates ranging from 14.3% (oculomotor and trochlear nerves) to 26.8% (facial nerve). There was also a predictable high rate of pyramidal involvement (38.9%).

The enhanced precision of intraoperative monitoring (IOM) makes both surgery and biopsy safer. Monitoring of corticobulbar function with corticobulbar motor evoked potentials (coMEPs) allows for the online monitoring of the functional integrity of the cranial motor nerves and the detection of early brainstem ischemia caused by occlusion of perforating arteries, which can lead to severe and irreversible neurological deficits [25].

Future investigation should aim at validating the effect of surgery assisted by IOM in a systematic, quantitative manner to provide a precise onco-functional balance.

### 4.5. Limitations

The primary limitations of adult BSG case series are the small and varied sample sizes in each study. Additionally, there is inconsistency in the selection of surgical approach (resection versus biopsy) between centers and obtaining raw data on the extent of resection achieved in different histological subgroups is unfortunately not possible. Most investigations included in this meta-analysis did not stratify overall survival (OS) data between various EOR groups. Therefore, it was not possible to aggregate data to demonstrate time differences in survival among different degrees of resection.

The case series results were not homogeneous for the inclusion of tumors with different biological behavior or for varied treatment regimens and schedules adopted after surgery.

Furthermore, there were no quantitative measures of residual neurological function following treatment, which precluded any analysis of functional outcomes. Additionally, given the period of data collection (1973–2011), most of the studies included patients with grade III and IV gliomas, using only histological classification without stratifying survival outcomes based on molecular profiling. This has the potential to produce confusing results. All the studies analyzed were published before the introduction of the WHO 2016 and WHO 2021 classification systems, which may introduce a crucial prognostic bias.

### 4.6. Future Perspectives

The rarity of brainstem gliomas (BSGs) and the heterogeneity of existing studies make it challenging to determine the optimal balance between oncological efficacy and functional outcomes in adult BSG patients. While surgical resection may confer a slight survival benefit in select cases, the higher risk of postoperative deficits must be weighed against the advantages in overall survival. As molecular profiling continues to advance, biopsy is becoming increasingly important in cases where surgery is not a safe option. Moving forward, an integrated analysis based on volumetric data and molecular–genetic profiles will be essential for identifying different categories of responders to specific treatment protocols, especially considering the 2021 WHO classification. Advances in cell-free plasma DNA techniques and molecular diagnosis at the single-cell level using liquid biopsy hold promise for revolutionizing BSG management by allowing diagnosis, prognosis, and response to postoperative treatment to be assessed before surgery. Given these innovations, a specialized multidisciplinary approach will be necessary for BSG management, leading the way for “Centers of Excellence” with appropriate technological resources and workloads.

## Figures and Tables

**Table 1:** Main demographical, anatomopathological, and surgical characteristics.

Article	Number of Patients	Age Median (m)	Low Grade	High Grade	Multifocal	Resection	GTR	STR	Biopsy	RT	Chemotherapy	OS HGG	OS Surgery HGG	OS Biopsy HGG	OS LGG
Babu et al., 2014 [7]	34	42.5	0	34	12	11	NA	NA	23	33	33	25.80	42.10	22.00	NA
Dey et al., 2014 [13]	240	48.7	0	240	NA	43	16	27	54	201	NA	7.00	9.00	NA	NA
Doyle et al., 2019 [14]	103	NA	0	103	NA	88	20	68	15	64	NA	11.53	12.14	8.00	NA
Kesari et al., 2008 [17]	101	36	31	15	31	11	NA	NA	45	82	40	16.80	NA	NA	124.13
Liu et al., 2021 [15]	256	NA	204	52	NA	187	134	53	69	128	52	NA	NA	NA	NA
Mursch et al., 2005 [16]	14	30	10	4	11	12	1	11	2	6	0	15.75	17.00	12.00	71.70
Total sample	748	44.19	32.8%	59.9%	36.2%	46.8%	27.9%	25.6%	27.9%	68.7%	30.9%	9.99 *	13.6	6.9	111.3

* Data from Dey et al. [13] and Kesari et al. [17] were excluded from median OS calculation due to absence of data regarding OS both in surgery and biopsy subgroups.

**Table 2:** Complication rates after biopsy or surgical resection.

	Biopsy n (%)	Notes	Resection n (%)	Notes
Babu et al., 2014 [7]	0/23 (0)	NA	2/11 (18.18)	1 cranial nerve palsy; 1 CSF leak
Kesari et al., 2008 [17]	4/14 (28.57)	Intracerebral bleeding; postop diplopia, paresis, and decreased speech; decreased upgaze; decreased mental status; subgaleal hematoma	5/14 (35.71)	Neurogenic pulmonary edema; decreased motor and cerebellar function; increased tone and rigidity
Mursch et al., 2005 [16]	0/2 (0)	NA	6/12 (50)	4 tetraparesis; 1 swallowing; 1 Bell’s palsy
Overall sample	4/39 (10.25)		13/37 (35.13)	

## Data Availability

All data generated or analyzed during this study are included in the published paper (and its supplementary information files).

## Supplementary Materials

The following supporting information can be downloaded at: https://www.mdpi.com/article/10.3390/curroncol30110709/s1, Table S1. Excluded studies; Table S2. Main demographical and clinical characteristics; Table S3. Main radiological, anatomopathological, and surgical characteristics; Figure S1. ROBINS-I tool was applied to evaluate the risk of bias (RoB); Figure S2. Funnel plot–hazard ratio (HR).

## Abbreviations

BSGs = brainstem glioma; CI = confidence interval; CNS = central nervous system; CT = chemotherapy; EOR = extent of resection; GTR = gross-total resection; HGG = high-grade glioma; HR = hazard ratio; LGG = low-grade glioma; MRI = magnetic resonance imaging; OS = overall survival; PFS = progression-free survival; PRISMA = preferred reporting items for systematic reviews and meta-analyses; RoB = risk of bias; RT = radiotherapy; SE = standard error; STR = subtotal resection; WHO = World Health Organization.
