# Supplementary material for: Surgical Management of Adult Brainstem Gliomas: A Systematic Review and Meta-Analysis
Source: Curr Oncol. 2023 Nov 7;30(11):9772–85. doi: 10.3390/curroncol30110709 (PMC10670486; doi:10.3390/curroncol30110709)
Supplement: Supplementary file 1 [file curroncol-30-00709-s001.zip › curroncol-2700688-supplementary.pdf]

Table S1. Excluded studies.

Cause for exclusion	Author and date of publication	
**Combined result without segregation of outcomes**	Babu 2013<br>Faulkner 2021<br>Kazunari 1999<br>Mohme 2018	Pollack 1996<br>Strauss 2013<br>Ueoka 2009<br>Xu 1997
**Case reports**	Dono 2020<br>Ferroli 2015	
**No surgery**	Dudoit 2021<br>Kandregula 2022<br>Leibtseder 2021	Reithmeier 2014<br>Salmaggi 2008
**Hazard Ratio not reported**	Guillamo 2001<br>Hundsberger 2014<br>Landolfi 1998<br>Lapras 1994<br>Majchrzak 2018<br>Meyronet 2017	Rigamonti 2021<br>Salvapadian 1999<br>Tasic 2016<br>Theeler 2015<br>Wang 2000<br>Waquar 2014
**Reviews**	Eisele 2016	

Table S2. Main demographical and clinical characteristics m =months.

Total Sample.	Mursch et al., 2005 [16]	Liu et al., 2021 [15]	Kesari et al., 2008 [17]	Doyle et al., 2019 [14]	Dey et al., 2014 [13]	Babu et al., 2014 [7]	Article
1973-2015	1986-1997	2006-2015	1998-2005	1973-2015	1973-2008	1998-2011	**Sample Date**
748	14	256	101	103	240	34	**Number of patients**
44.19	30	NA	36	NA	48,7	42,5	**Age median (m)**
17-79	17-66	NA	18-79	NA	NA	18-71	**Age range (m)**
48.98%	11	NA	50	59	102	19	**Gender (M)**
12.08	12	NA	4	NA	NA	2	**Time before presentation (m)**
14.3%	2	NA	NA	NA	NA	NA	**CN III**
14.3%	2	NA	NA	NA	NA	NA	**CN IV**
35.7%	5	NA	NA	NA	NA	NA	**CN VI**
22.1%	6	NA	22	NA	NA	5	**CN V**
26.8%	7	NA	25	NA	NA	8	**CN VII**
16.1%	4	NA	12	NA	NA	8	**CN VIII**
28.7%	10	NA	23	NA	NA	NA	**CN IX,X,XI**
10.9 %	NA	NA	11	NA	NA	NA	**CN XII**
38.9%	10	NA	42	NA	NA	6	**Pyramidal Symptoms**
31.1%	NA	NA	40	NA	NA	2	**Sensory loss**
39.6%	7	NA	38	NA	NA	14	**Cerebellar**
18.8%	NA	NA	19	NA	NA	NA	**Dysphagia**

Table S3. Main radiological, anatomopathological and surgical characteristics.

Total sample	Mursch et al., 2005 [16]	Liu et al., 2021 [15]	Kesari et al., 2008 [17]	Doyle et al., 2019 [14]	Dey et al., 2014 [13]	Babu et al., 2014 [7]		Article
34.9%	4	NA	43	NA	NA	5	**Mid brain**	**Localization**
63.8%	12	NA	65	NA	NA	18	**Pons**	
47.0%	9	NA	52	NA	NA	9	**Medulla**	
36.2%	11	NA	31	NA	NA	12	**Multi focal**	
9.8%	1	56	16	0	0	0	**WHO Grade I**	**WHO Classi-fication**
23.0%	9	148	15	0	0	0	**WHO Grade II**	
20.7%	3	20	12	23	75	22	**WHO Grade III**	
36.5%	1	32	3	60	165	12	**WHO Grade IV**	
32.8%	10	204	31	0	0	0	**Low Grade**	
59.9%	4	52	15	103	240	34	**High Grade**	
46.8%	12	187	11	88	43	11	**Resection**	**Treatment**
27.9%	1	134	NA	20	16	NA	**GTR**	
25.6%	11	53	NA	68	27	NA	**STR**	
27.9%	2	69	45	15	54	23	**Biopsy**	
68.7%	6	128	82	64	201	33	**RT**	
30.9%	0	52	40	NA	NA	33	**Chemo theraphy**	

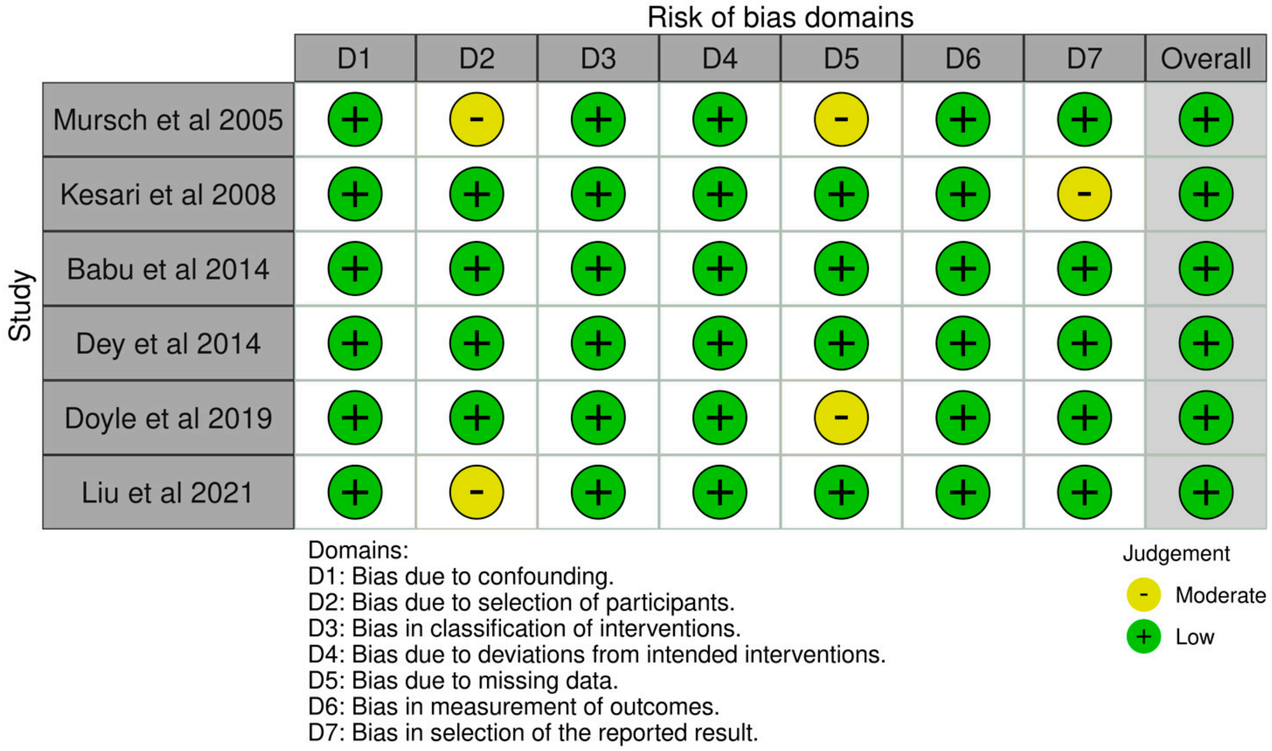

Figure S1. ROBINS-I tool was applied to evaluate the Risk of Bias (RoB) [7, 13–17]**.** All the included studies were retrospective single center and were rated " moderate" to "low" risk of bias.

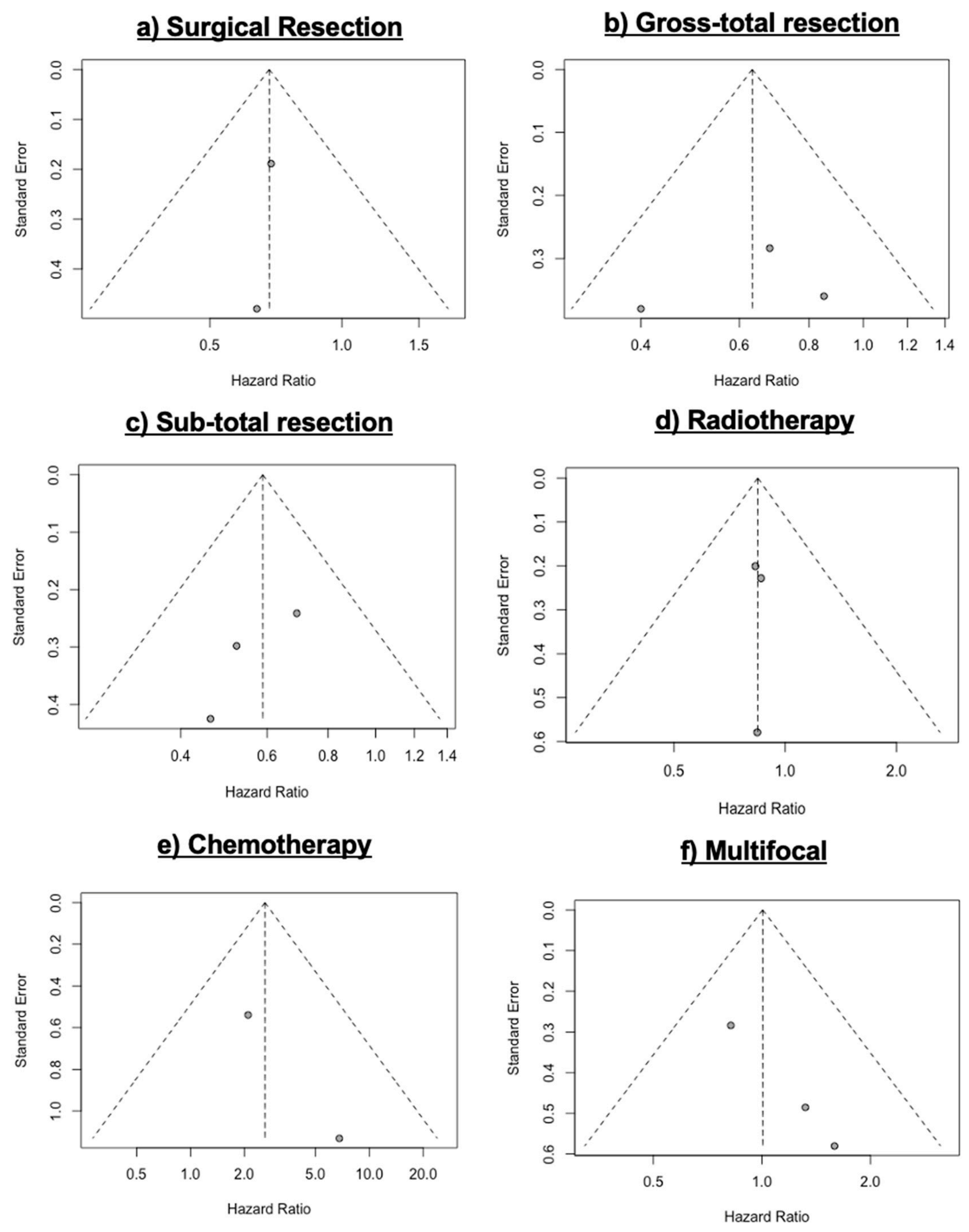

Figure S2. Funnel plot-hazard ratio (HR) The distribution of HR and standard error of all included studies demonstrating level of precision among studies in investigating effectiveness of surgical resection, radiotherapy, chemotherapy and multifocal presentation for the treatment of brainstem gliomas.
